# Seed dormancy release accelerated by elevated partial pressure of oxygen is associated with *DOG* loci

**DOI:** 10.1093/jxb/ery156

**Published:** 2018-04-26

**Authors:** Gonda Buijs, Jan Kodde, Steven P C Groot, Leónie Bentsink

**Affiliations:** 1 Wageningen Seed Laboratory, Laboratory of Plant Physiology, Wageningen University, Wageningen, Netherlands; 2 Wageningen Plant Research, Wageningen University and Research Centre, AP Wageningen, The Netherlands

**Keywords:** After-ripening, *Arabidopsis thaliana*, *Delay of Germination*, *DOG*, elevated partial pressure of oxygen, EPPO, seed dormancy, quantitative trait loci

## Abstract

Seed dormancy determines the timing of seed germination and may be released by dry storage, also referred to as after-ripening. Studies on dormancy-release mechanisms are often hampered by the long after-ripening requirements of seeds. After-ripening is thought to be mainly caused by oxidative processes during seed dry storage. These processes are also the main cause of seed ageing. Increasing partial oxygen pressure through the elevated partial pressure of oxygen (EPPO) system has been shown to mimic and accelerate dry seed ageing. In this study, we investigated whether the EPPO system may also release primary seed dormancy in *Arabidopsis thaliana*. EPPO mimics dry after-ripening at the genetic level, as quantitative trait locus (QTL) analysis after EPPO treatment identified the *DELAY OF GERMINATION* loci *DOG1*, *DOG2*, and *DOG6* that were first described in a study using dry after-ripening to release seed dormancy. QTL analysis also showed that dormancy release by cold stratification (another common method to break seed dormancy) partly overlaps with release by after-ripening and EPPO treatment. We conclude that EPPO is an appropriate method to mimic and accelerate dormancy release and, as such, may have applications in both research and industry.

## Teaser

Storage of seeds under elevated partial pressure of oxygen mimics dry after-ripening at the genetic level, as indicated by identification of *DELAY OF GERMINATION* quantitative trait loci.

## Introduction

The seed is the unit for propagation, dispersal, and survival of seed plants. Orthodox seeds can withstand drying and can thus survive over time and be dispersed over distance. The seed has to germinate and establish a seedling in order to grow and finally propagate. The timing of germination is essential for successful seedling establishment. As plants in temperate regions often disperse their seeds in autumn, immediate germination of the seed would cause it to grow in winter, decreasing the chance of seedling survival and propagation. Therefore, most species have dormancy mechanisms that control the timing of germination. A viable seed possesses dormancy when it is temporarily unable to germinate under favourable conditions. Dormancy induced during seed maturation is called primary dormancy, and this can be caused by physiological, physical, or developmental factors, or by a combination of these (Baskin and Baskin, 2004; Bewley *et al.*, 2013). *Arabidopsis thaliana* displays coat-enhanced physiological dormancy in which the balance between the hormones abscisic acid (ABA; germination-inhibiting) and gibberellins (GAs; germination-promoting) is key in determining the germination status (Baskin and Baskin, 2004; Bewley *et al.*, 2013). However, this hormonal balance is the result of multiple complex pathways and processes, of which the underlying mechanisms largely remain unknown (Nonogaki, 2014; Dekkers and Bentsink, 2015; Chahtane *et al.*, 2017). Seed dormancy has been studied for both scientific reasons (e.g. the intriguing dormant stage in a diploid life stage) and for agricultural reasons (e.g. rapid germination for crops). In the laboratory, there are multiple methods to relieve seed dormancy and to assess and quantify the dormancy level of seed batches. One method is to expose the seeds to a period of cold imbibition (cold stratification, CS; Finch-Savage *et al.*, 2007). In Arabidopsis, stratification induces the expression of gibberellic acid biosynthesis genes and the resulting high levels of GAs release dormancy (Yamauchi *et al.*, 2004). Another common method to relieve and assess dormancy is to store the seeds under dry conditions, so-called dry after-ripening (AR). The period of dry storage that is required to release dormancy is often expressed as days of seed dry storage to reach 50% of germination (DSDS_50_; Alonso-Blanco *et al.*, 2003). In true potato seeds, AR can be accelerated when the seeds are stored dry under an elevated temperature (37 °C; Alvarado and Bradford, 2005). Dry dormancy release is most likely caused by the formation and action of reactive oxygen species (ROS) (Oracz *et al.*, 2007; El-Maarouf-Bouteau and Bailly, 2008; El-Maarouf-Bouteau *et al.*, 2013; Morscher *et al.*, 2015). The production of ROS has been shown to occur both in dry and imbibed seeds of Arabidopsis (Leymarie *et al.*, 2012), barley (Ma *et al.*, 2016), and sunflower (Oracz *et al.*, 2007). ROS can potentially react with all molecules in a cell, such as lipids, DNA and RNA, proteins, and carbohydrates, and oxidation of a molecule may change its functioning. Oxidized proteins are damaged and degraded upon seed imbibition (reviewed by El-Maarouf-Bouteau *et al.*, 2013; Morscher *et al.*, 2015). The degradation of specific proteins might end the inhibition of germination, either directly by the removal of the proteins or indirectly by inducing germination-promoting signalling pathways. Oxidation of mRNAs has been reported to be important for dormancy release in sunflower seeds (Bazin *et al.*, 2011); however, the underlying molecular mechanisms remain elusive (reviewed by Nonogaki, 2014). Oxidation not only results in dormancy release but also causes seed ageing. These processes are hard to separate (Morscher *et al.*, 2015). Ageing probably starts directly after seed dispersal (or already during seed maturation). First it results in the release of seed dormancy and, later, in seed deterioration as ROS accumulate during seed ageing (Bailly *et al.*, 2008). In accordance with this, seed storage under anoxia slows down seed ageing (Groot *et al.*, 2015). Based on this role of oxygen, a method has been developed to mimic and accelerate dry seed ageing, namely elevated partial pressure of oxygen (EPPO) storage (Groot *et al.*, 2012). In the EPPO method, seeds are stored dry under ambient air, but under increased pressure. This increases the absolute amount and partial pressure of oxygen (pO_2_) in the storage environment. During both dry ageing and EPPO storage seed tocopherol levels decrease, a process that does not occur under controlled deterioration [storage under high relative humidity (RH) and high temperatures; Groot *et al.*, 2012]. Moreover, EPPO seems to mimic natural ageing better than controlled deterioration in barley, based on phenotypic (e.g. normal seedling formation) and quantitative trait locus (QTL) analyses (Nagel *et al.*, 2016). To identify loci that affect dry after-ripening, genetic approaches have been used in, for example, weedy rice (Gu *et al.*, 2004), barley (Sato *et al.*, 2009), and sorghum (Guo *et al.*, 2015). In Arabidopsis, QTL analysis for dry after-ripening requirement, expressed as DSDS_50_, resulted in the identification of eleven *DELAY OF GERMINATION* (*DOG*) loci in a combined mapping using six recombinant inbred line (RIL) populations (Bentsink *et al.*, 2010). The RIL population of the Landsberg *erecta* and Cape Verde Island accessions (L*er*/Cvi) has led to the identification of the most significant QTLs (Alonso-Blanco *et al.*, 2003; Bentsink *et al.*, 2010). The *DOG* QTLs identified in the L*er*/Cvi RIL population have been confirmed by near-isogenic lines (NILs) in which the QTL regions from the Cvi or Kashmir-2 accession were introgressed into the L*er* genotype (Alonso-Blanco *et al.*, 2003; Bentsink *et al.*, 2010). These NILs have been used to identify the genes underlying the *DOG* QTLs. Thus far, only the causal genes for *DOG1* (Bentsink *et al.*, 2006) and *DOG18* have been identified (Xiang *et al.*, 2016). These studies show the power of the use of genetic populations in understanding the regulation of quantitative traits.

Here, we investigated whether EPPO may release primary seed dormancy. Dormancy release by AR often takes a long time, for example, more than a year for very dormant accessions of Arabidopsis (Vidigal *et al.*, 2016). Accelerating this process is beneficial for both seed dormancy research and commercial applications. We show that EPPO accelerates dormancy release in L*er* and the very dormant *DOG1* NIL. EPPO mimics dry after-ripening very well, as shown by the large overlap of DSDS_50_ and EPPO QTLs in the L*er*/Cvi RIL population. The identified QTLs were confirmed by testing a set of *DOG* NILs. Finally, the results are compared and discussed in relation to dormancy QTLs identified by cold stratification.

## Material and methods

### Plant material

Seeds of the *Arabidopsis thaliana* L*er* accession, NIL*DOG1*-Cvi, NIL*DOG2*-Cvi, NIL*DOG3*-Cvi, and NIL*DOG6*-Kas-2 in the L*er* background, and the L*er*/Cvi RIL population were used, previously described by Bentsink *et al.* (2010) and Alonso-Blanco *et al.* (1998), respectively. The NIL set was grown on Rockwool in 2016 and the L*er*/Cvi RIL population was grown in soil in 2007 in a greenhouse under a 22 °C and a 16/8 h light/dark regime. All plants were grown with three biological replicates. After harvest the seeds were stored at –80 °C until use. Prior to the start of the experiments approximately 500 seeds were taken from the –80 °C freezer and placed in open 1.5-ml screw-cap tubes. Subsequently, the open tubes were placed at 20 °C and 35% RH for 3 d. During this acclimation period, the seeds were exposed to air and thus experienced a brief period of AR. Three biological replicates of the NIL set were used in all experiments. For the RIL population, only one biological replicate was used in the EPPO experiment, and another biological replicate was used in the stratification experiment. For the EPPO and the stratification experiments, respectively, 152 and 134 lines of the L*er*/Cvi RIL population were used (Supplementary Table S1 at *JXB* online). The parental lines were also included.

### AR storage

The AR data that we used originated from Alonso-Blanco *et al.* (2003). These seeds had been stored at ambient conditions with limited temperature control and no humidity control.

### EPPO storage

During the 3-d acclimation period, sub-samples of approximately 50 seeds were taken and placed into 1.5-ml screw-cap tubes. Two holes were punctured in the screw-cap, the rubber ring was removed, and a piece of filter paper was placed inside the screw-cap to prevent the seeds from spilling through the holes. For each storage period one 1.5-l steel tank was used, into which the screw-cap tubes containing the 152 RILs and both parents were placed. A total of four tanks were used, and each was filled with compressed air as described by Groot *et al.* (2012). To set the relative humidity in the tanks to 35%, a nylon stocking with silica gel equilibrated at 35% RH was added to each one. All tanks were filled over 25 min with air to a pressure of 8 MPa. The tanks were placed at 20 °C for 34 d for the RIL population and for 29 d for the NIL set. For the NIL set, the pressure was subsequently increased to 20 MPa for 12 d. Control seed samples were stored in an air-tight jar at 20 °C and 35% RH. For the N_2_ treatment, tanks were flushed with N_2_ prior to filling in order to remove residual air. To test the effect of the rate of pressure build-up, two N_2_-filled tanks were filled either quickly (0.1 MPa to 8 MPa in 2.5 min) or slowly (0.1 MPa to 8 MPa in 25 min). Pressure release was controlled to prevent physical damage to the seeds that might be caused by a too-rapid expansion of gasses present in the intercellular spaces, using computer-controlled flow control equipment such that the relative pressure decline was maintained at 0.5% per minute.

### Germination and viability assays

For all germination experiments, seeds were sown on blue germination paper in trays with 48 ml demineralised water and placed in a cabinet at 22 °C with continuous light. Each tray contained six samples of approximately 50 seeds. Seed germination was followed for 5 d using the Germinator system (Joosen *et al.*, 2010). Viability of the non-germinated seeds was checked by placing the seeds in a new germination tray with 10 mM KNO_3_ added to the demineralised water. After 1 d in nitrate the seed coat was removed from the remaining non-germinated seeds. At 2 d after seed-coat removal, viability was assessed by checking for growth of the embryo (greening of the cotyledons and radicle elongation).

### Stratification experiment

The seeds were sown as described for the standard germination experiment above. Seeds were taken from the –80° freezer and stored at 20 °C and 35% RH for 4 d prior to the stratification experiment. After sowing, the seeds were placed in a cold room at 4 °C for 10, 8, 6, 4, 2, or 0 d. Thus, prior to stratification the seeds were stored dry on the bench for 4, 6, 8, 10, 12, or 14 d, respectively. After cold storage all trays, including non-stratified seeds after 14 d of bench storage, were placed simultaneously in a germinator incubator at 22 °C as described.

### Analysis of DOxy_50_, DC_50_, and quantitative trait loci

The days of seed EPPO storage to reach 50% germination (DOxy_50_), days of seed cold storage to reach 50% germination (DC_50_) and days of dry seed storage to reach 50% germination (DSDS_50_) were calculated using the statistical program R version 2.14 (R Development Core Team, 2009; www.r-project.org) according to He *et al.*, 2014). QTL analyses were performed with the MapQTL program (version 6, www.kyazma.nl; van Ooijen, 1992). QTLs were identified with both interval mapping and rMQM mapping according to the manual. See Supplementary Table S1 for the phenotypic data that was used.

## Results

### Storage under EPPO conditions releases seed dormancy

**Fig. 1.**
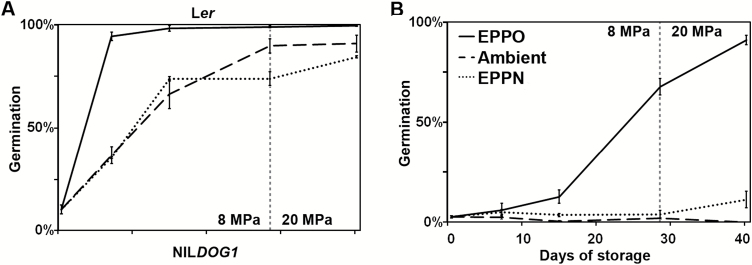
Dormancy release under elevated partial pressure of oxygen (EPPO) conditions. Germination percentages of L*er* (A) and NIL*DOG1* (B). The seeds were germinated after 0, 6, 14, or 28 d of treatment: EPPO at 8 MPa, ambient conditions, N_2_ at 8 MPa (EPPN). After 28 d, the pressure was increased from 8 to 20 MPa for both the EPPO and EPPN treatments (indicated by the vertical dotted line) and the final germination assay was performed after 12 d of storage at 20 MPa. Error bars represent s.e.m., *n*=3.

To investigate whether EPPO can release primary seed dormancy in Arabidopsis, a preliminary test was performed. Fresh seeds of the deeply dormant NIL*DOG1* genotype were stored for 2 weeks under 3, 6, 9, or 12 MPa of air, with ambient air (0.1 MPa) as a control (Supplementary Fig. S1). Storage for 2 weeks at 3 and 6 MPa did not significantly reduce dormancy levels in comparison to ambient air storage. Storage at 9 and 12 MPa resulted in dormancy release, but this but the resolution of the test was not sufficient to be able to identify temporal changes in low-dormant genotypes. To obtain better temporal resolution and a full dormancy release we tested EPPO at 8 MPa for 0–28 d followed by a short period (12 d) of EPPO at 20 MPa with dormant NIL*DOG1* and L*er* seeds (Fig. 1). This protocol allowed a gradual dormancy release for both the low- (Fig. 1A) and deep-dormant genotypes (Fig. 1B) and eventually resulted in 100% germination. To investigate whether the effect of EPPO was due to the elevated pO_2_, a treatment with 8 MPa of pure nitrogen gas (N_2_) was performed (Fig. 1); similar to the EPPO air treatment, the pressure was increased to 20 MPa after 28 d of storage at 8 MPa. The N_2_ treatment with no free oxygen under 8 and 20 MPa showed no significant difference compared to ambient storage. To exclude potential effects of the pressure itself, we compared the effect of the rate of pressure build-up. Seeds were stored under 8 MPa of N_2_, with a fast (2.5 min) or slow (25 min) build-up of pressure Fig. S2A. There was no effect observed for the rate of pressure build-up itself compared to ambient storage (Fig. S2B). The EPPO treatments as performed in our experiments did not result in visually aged seeds, in that we did not observe morphologically aberrant seedlings, e.g. stunted root growth or discoloration of the cotyledons, which are the first signs of seed ageing in cruciferous species (ISTA, 2018).

### EPPO mimics dry seed after-ripening

**Fig. 2.**
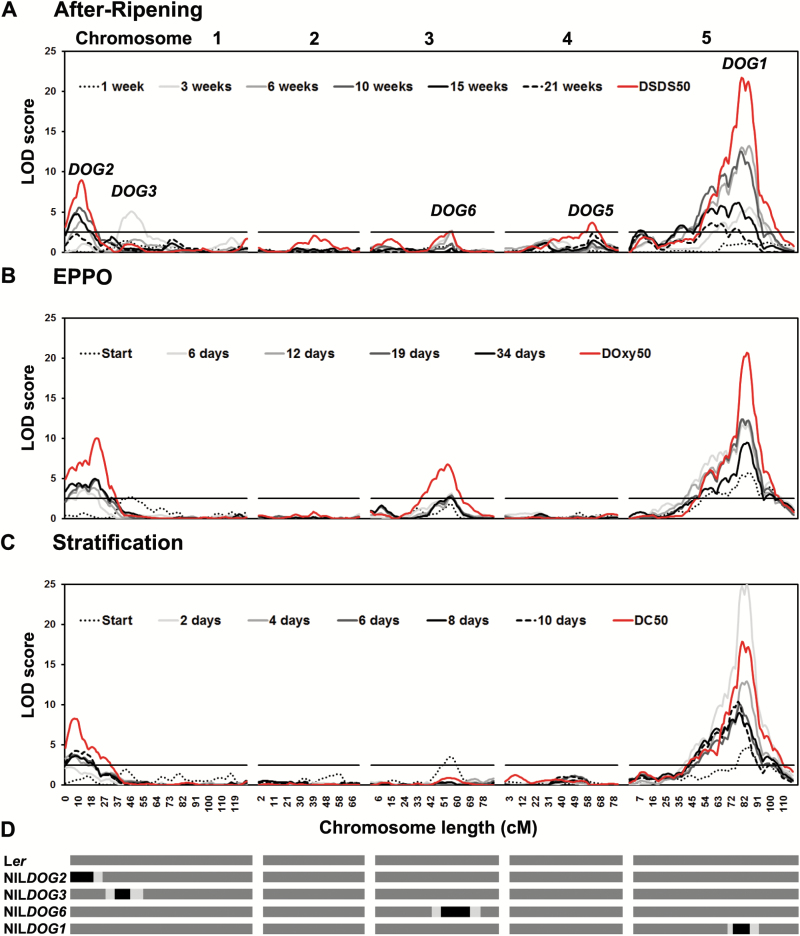
QTL mapping of dormancy release during after-ripening (AR), elevated partial pressure of oxygen (EPPO) treatment, and cold stratification (CS). Logarithm of the odds ratio (LOD) score maps of dormancy release after AR (A), EPPO (B), and CS (C) for the five chromosomes of Arabidopsis. The graphs show interval mapping of the germination percentages after the different storage periods (grey and black lines) and rMQM mapping of (A) DSDS_50_, (B) DOxy50, and (C) DC_50_ (red lines). The horizontal black lines represent the LOD score threshold above which a QTL is significant (LOD=2.5, *P*<0.05). (D) Graphical representation of the Cvi introgression in the NIL*DOG* genotypes. Dark grey represents the L*er* background and black represents the Cvi introgression, flanked by light-grey regions of the introgression recombination breakpoints.

If EPPO dormancy release mimics dormancy release by dry AR, we would expect to identify the same loci when performing QTL analyses. The L*er*/Cvi RIL population had previously been used to investigate the genetic basis of seed dormancy. In those experiments QTL mapping for after-ripening requirement was performed on the germination percentages after each storage period on the laboratory bench (1, 3, 6, 10, 15, and 21 weeks of dry AR) and on the DSDS_50_ value that was derived from these germination percentages (Fig. 2A; data from Alonso-Blanco *et al.*, 2003). These analyses led to the identification of *DOG1*, *DOG2*, *DOG5*, and *DOG6*. We used this same population to investigate seed dormancy release after EPPO storage. Germination percentages after various intervals of EPPO storage at 8 MPa were determined (Supplementary Fig. S3B). With the same method used to calculate the DSDS_50_ (Alonso-Blanco *et al.*, 2003), the days of EPPO storage to reach 50% of germination (DOxy_50_) were calculated (Fig. S3D). DOxy_50_ showed a strong correlation with DSDS_50_ (Pearson’s *r*=0.72, Fig. S3E). Furthermore, after correction for the high number of low-dormant lines in the L*er*/Cvi RIL population (Fig. S3D) the correlation remained high (Pearson’s *r*=0.94, Fig. S3E). QTL analyses were performed for DOxy_50_ and for the germination percentages after each storage interval (0, 6, 12, 19, and 34 d, Fig. 2B). QTL analysis for the EPPO treatment (DOxy_50_) identified the *DOG1*, *DOG2*, and *DOG6* QTLs, which explained 59.3% of the variance. The variance explained by the four DSDS_50_ QTLs was 62%. The *DOG1*, *DOG2*, and *DOG6* regions overlapped with the previously identified DSDS_50_ QTL, which indicated that EPPO dormancy release mimicked dry after-ripening.

**Fig. 3.**
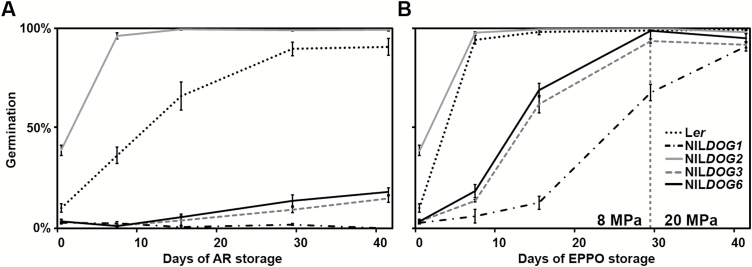
Dormancy release by elevated partial pressure of oxygen (EPPO) and after-ripening (AR). Germination percentages of dormant L*er* and *DOG* NILs at different intervals during AR (A) and EPPO treatment (B). After 29 d of EPPO at 8 MPa the pressure was increased to 20 MPa (indicated with vertical dotted line), and the germination was assessed again after a further 12 d. Error bars represent s.e.m., *n*=3.

The effect of the QTLs was confirmed by the use of the *DOG* NILs that contain Cvi introgression fragments in a L*er* background at the position of the QTL (Fig. 2D). The NILs showed the same trend in dormancy release dynamics under both AR and EPPO conditions, but EPPO dormancy release was much quicker (Fig. 3). In both conditions the NIL*DOG2* genotype released dormancy most rapidly, closely followed by L*er*. Dormancy was released at the lowest rate in both treatments in NIL*DOG1*, followed by NIL*DOG3* and NIL*DOG6.* Overall, EPPO dormancy release mimicked AR dormancy release on the genetic level.

### QTLs for dormancy release by cold stratification largely overlap with AR and EPPO QTLs

Cold stratification (CS) is another method used to assess the dormancy level (Yamauchi *et al.*, 2004; Penfield and Springthorpe, 2012). The L*er*/Cvi population was sown and stratified for 0, 2, 4, 6, 8, or 10 d and the days of cold storage required to reach 50% of germination (DC_50_) was calculated. QTL mapping of dormancy release by CS in the L*er*/Cvi RIL population was performed to investigate whether the same loci were identified as for dormancy release by AR. Based on the DC_50_, both *DOG1* and *DOG2* QTLs were identified (Fig. 2C). The DC_50_ QTL explained 56.4% of the total variance. The DC_50_ mapping lacked the *DOG6* locus, which was identified in the DOxy_50_ and DSDS_50_ mappings. However, QTL mapping based on the germination percentages at the start of the CS treatment revealed that the *DOG6* QTL was identified before stratification (Fig. 2C). Thus, the *DOG6* locus is either very sensitive to stratification, indicating that *DOG6* dormancy is efficiently removed by CS and therefore no allelic variation is detected, or there is no allelic variation for the response of *DOG6* to stratification.

**Fig. 4.**
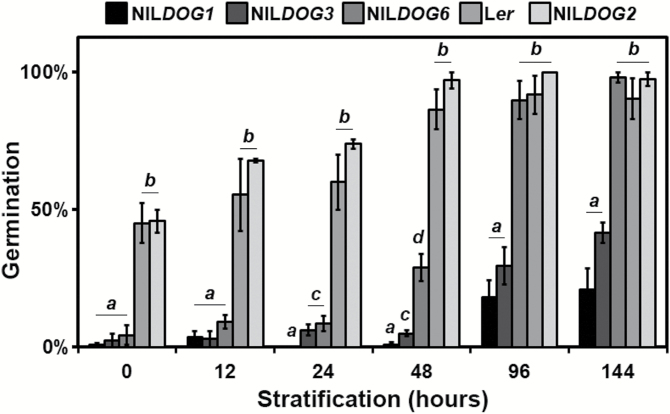
Germination behaviour of dormant *DOG* NIL and L*er* seeds after different stratification periods at 4 °C. Different letters indicate genotypes that have significantly different germination percentages at each time-point (Student’s *t*-test, *P*<0.05). Error bars represent s.e.m., *n*=3.

To further investigate the stratification requirement of the *DOG* NILs, germination was determined after 0, 12, 24, 48, 96, or 144 h of cold stratification. The less-dormant genotypes L*er* and NIL*DOG2* released dormancy quickly during CS treatment, whereas the more-dormant genotypes NIL*DOG1* and NIL*DOG3* released dormancy more slowly (Fig. 4). NIL*DOG6* showed an initial slow rate of dormancy release, similar to NIL*DOG1* and NIL*DOG3* (after 12 and 24 h of stratification). However, the rate of NIL*DOG6* dormancy release suddenly increased between 24 and 48 h of stratification, resulting in significantly different germination as compared with both the high- and low-dormant NILs.

## Discussion

Although it has been the subject of considerable research interest, knowledge of the genetic and molecular mechanisms of seed dormancy is still limited (reviewed by Née *et al.*, 2017). Genetic approaches using natural variation in Arabidopsis have identified the *DOG* loci (Alonso-Blanco *et al.*, 2003); However, only a few genes underlying these QTLs have been identified, among which is the major seed dormancy regulator *DOG1*. The protein levels of DOG1 correspond to the primary dormancy levels (Nakabayashi *et al.*, 2012). Interestingly, DOG1 protein levels do not decrease as dormancy is released, indicating that its activity is altered during dry storage. This alteration of DOG1 is thought to be caused by oxidation (Nakabayashi *et al.*, 2012). To study dormancy and the role of oxidation, dormancy release can be monitored during seed dry storage (AR), but this can last a long time. A well-known example of an Arabidopsis accession with a high AR requirement for dormancy release is Cvi (Finch-Savage *et al.*, 2007), but this is a characteristic that is not limited to just a few accessions. The Iberian Population, for example, is very dormant as a whole, requiring up to 559 d of dry storage to reach 50% germination (Vidigal *et al.*, 2016). To be able to both accelerate and mimic dry AR, we used the EPPO method. During EPPO treatment, the relative amount of oxygen was the same as under ambient air pressure but the pO_2_ was increased: under EPPO treatment at 8 MPa the pO_2_ was 1.68 MPa as compared to 0.021 MPa under ambient conditions. Here, we showed that EPPO treatment released dormancy quickly and in a controlled manner, while the seeds remained dry. EPPO mimicked dormancy release under dry AR at the genetic level, as shown by comparing the QTLs identified for EPPO dormancy release (*DOG1*, *DOG2*, and DOG*6*) with those identified for AR requirement (*DOG1*, *DOG2*, *DOG6*, and *DOG5*; Alonso-Blanco *et al.*, 2003). This supports the hypothesis that dormancy release by AR is mainly caused by oxidative processes (Oracz *et al.*, 2007; El-Maarouf-Bouteau and Bailly, 2008; El-Maarouf-Bouteau *et al.*, 2013; Morscher *et al.*, 2015). This is certainly true for DOG1, the protein that underlies the *DOG1* QTL (Nakabayashi *et al.*, 2012). The *DOG5* locus that was identified based on the DSDS_50_ analysis was the only QTL not identified in the DOxy_50_ QTL mapping (Fig. 2A, B). A possible explanation for this is the difference in RH between the AR and EPPO storage conditions. Dormancy release is known to be influenced by the moisture content (Probert, 2000). The *DOG* QTLs identified previously were identified based on dry AR in ambient conditions (estimated humidity fluctuated between 40 and 65%). During EPPO storage, the RH was constantly low (35%) and small differences in moisture content might have large effects during storage (Labuza, 1971). The lack of RH fluctuations during the EPPO treatment might also explain why the EPPO QTL mapping displayed such a high explained variance, even though only one biological replicate was used. Apart from the RH fluctuations that might occur under non-RH controlled AR storage, temperature fluctuations and other time-related factors were also eliminated in the EPPO treatment as compared to dry AR. DSDS_50_ and DOxy_50_ were calculated based on multiple germination assays after various storage intervals and thus provide a robust measure of dormancy level. However, the *DOG* loci also showed a temporal pattern during dormancy release (Alonso-Blanco *et al.*, 2003). For example, the *DOG2* locus could not be identified before 6 weeks of AR, but it was identified after all further AR storage periods (Fig. 2A). As these temporal patterns provide insights regarding the dormancy mechanisms underlying the different QTLs, we also studied and compared the *DOG* loci after each AR and EPPO storage period (Fig. 2A, B). To be able to know which AR storage periods were congruent with storage periods in EPPO, we analysed the G_max_ frequency distributions from the different AR and EPPO storage periods and performed correlation analyses on the G_max_ percentages (Supplementary Fig. S3A, B, F). These analyses indicated that 0 d in EPPO corresponded with 3 weeks AR, 6 and 12 d EPPO with 6 weeks AR, and 19 and 34 days EPPO with 10 weeks AR (Supplementary Fig. S3F). QTL analyses on these storage periods allowed a more detailed comparison of the *DOG* loci identified during AR and EPPO storage, and thus we could compare the genetic response to both treatments over storage time (Fig. 2, Supplementary Fig. S3G). The different temporal patterns indicated that the underlying mechanisms of dormancy release were different for the different loci. The *DOG2* locus was first identified after 6–12 d of EPPO and 6 weeks of AR storage, and was identified similarly in both treatments thereafter. This indicated that the underlying mechanism of the *DOG2* locus had a gradual response to oxidation. In contrast, the *DOG3* locus responded more quickly to oxidation, as it was only identified at 3 weeks of AR storage and prior to EPPO treatment, and not after longer storage periods under either treatment. This indicated that the underlying mechanism of the *DOG3* locus may be highly responsive to oxidation. With the exception of 1 week of AR, the *DOG1* locus was identified following any period of AR and EPPO storage. This corresponds with the hypothesis that the DOG1 protein is oxidized gradually over storage time (Nakabayashi *et al.*, 2012). Only the *DOG6* locus showed a different pattern, as it was identified earlier during AR storage but only from 6 d EPPO treatment onward. We have already noted that the QTL for *DOG6* is very sensitive to CS, as shown in Figs 2C and 4. This supports previous findings that dormancy release is regulated by multiple additive pathways (Bentsink *et al.*, 2010).

Identifying the precise location of the *DOG2* locus has proved challenging and is probably because of its close vicinity of the *DOG3* locus, which has an opposite effect on dormancy and which was not identified in the EPPO mapping. The EPPO storage showed this QTL to be slightly more to the middle of the chromosome compared to the AR analyses. However, the experiments with the NIL*DOG2* genotype confirmed the effect of EPPO on the *DOG2* locus. All the QTL data combined indicate that EPPO mimics AR storage.

We have not yet investigated how EPPO affects seed dormancy at the cellular or molecular level. However, a hypothesis is that proteins essential for the regulation of seed dormancy are either better protected against oxidation or more sensitive to oxidation, depending on their role. Protection could, for example, consist of cruciferin proteins, which have been suggested to buffer against oxidative stress (Nguyen *et al.*, 2015). The EPPO method allows detailed studies of the effects of oxidation on seed dormancy and longevity in a very controlled way, and it provides a quick method to remove dormancy from seeds batches. We also compared EPPO treatment with CS, another dormancy-releasing method. A significant difference with AR or EPPO is that during CS the seeds imbibe and are metabolically active, which allows processes such as translation and transcription. During dry ageing, at least at 35% RH, there is no measurable metabolic activity and enzymatic repair processes cannot take place (Labuza, 1971). An example of the difference between CS and AR is that some accessions from a natural population collected in the Iberian Peninsula barely release dormancy during dry AR, but do release dormancy after CS (Vidigal *et al.*, 2016). The difference in sensitivity to AR and CS might be explained by a difference in sensitivity to, or production of, GA. Physiological dormancy consists of multiple layers (e.g. sensitivity to nitrate, light, and temperature) and whether or not seeds will respond to GA depends on these layers. It is known that ABA levels first have to be low before GA can promote germination (Finch-Savage and Leubner-Metzger, 2006).

The *DOG1* and *DOG2* loci were the only two that were identified in all three analyses (DSDS_50_, DOxy_50_, and DC_50_). QTL mapping for DC_50_ did not reveal additional QTLs, nor did the QTL analyses on the individual stratification time-points (Fig. 2C). This suggests that dormancy release through CS is (partly) different from dormancy release through oxidation; however, this requires further research. Multiple tests were performed in order to establish that EPPO functioned through oxidative processes and not through the high pressure itself. First, dormancy release was studied under slow and quick pressure build-up (Supplementary Fig. S2A), and no significant differences were observed. The tanks were filled with N_2_ but not flushed, so oxygen concentrations were comparable to ambient conditions. We chose to use this non-flushed N_2_ treatment because under EPPO air conditions dormancy release might have been too quick to be able to measure the differences. Second, we studied dormancy release without the presence of gaseous oxygen (tanks flushed and filled with N_2_, Supplementary Fig. S2B). This treatment was performed with air (EPPO) as well as N_2_ under the same pressure (ambient and 8 MPa, Fig. 1, Supplementary S2B). Under the N_2_ treatment at 8 MPa (subsequently increased to 20 MPa), the seeds released dormancy at the same rate as under AR ambient conditions. Remarkably, under ambient 0% oxygen conditions (0.1 MPa N_2_) dormancy release occurred at the same rate as under AR for the period tested. After 15 and 41 d of treatment, there was a significant difference between L*er* stored under 0% oxygen at ambient and elevated pressure conditions (*P*<0.05). The fact that dormancy release did not stop completely without oxygen present in the tanks (flushed N_2_ treatments, Fig. 1, Supplementary S2B) can be explained by residual oxygen or ROS in the seeds. The pressure itself did not cause the dormancy release, as there was no observable effect when there was no gaseous oxygen present or when the rate of pressure build-up was increased. Abnormal seedling formation or rupture of the seed coat other than at the site of radicle protrusion was not observed after EPPO storage.

## Conclusions

EPPO is the first method for which it has been proved genetically that it mimics and accelerates AR. It provides a quick and reliable method to assess dormancy levels or to remove dormancy altogether in seed batches, and it allows the mechanisms underlying the control of seed dormancy to be studied. A big advantage of the EPPO method is that the seeds do not imbibe during the treatment, and this allows subsequent treatments, storage, or experiments to be carried out. EPPO has been applied to other species, including lettuce, soybean, and barley, and they have responded well to the method when it has been used for seed ageing (Groot *et al.*, 2012; Nagel *et al.*, 2016). The method has to be adapted for each different species, with the optimal pressure, RH, and temperature needing to be determined. Finally, as seed ageing and seed dormancy are intertwined, the EPPO dormancy release method can be combined with the EPPO ageing method to enable the complete seed life span to be studied in one experiment: the pressure can easily be increased after dormancy release to produce accelerated ageing EPPO conditions. Furthermore, temperature and humidity during storage can be controlled and varied.

## Supplementary data

Supplementary data are available at *JXB* online.

Fig. S1. Germination of dormant NIL*DOG1* seeds after different EPPO storage treatments.

Fig. S2. Effect of the rate of pressure build up on the dormancy release of L*er* and NIL*DOG1* seeds.

Fig. S3. Frequency distributions and correlation plots of the phenotypic data used for the QTL analyses.

Table S1. Phenotypic data for the L*er*/Cvi RILs used for the QTL analyses.

Supplementary Table

Supplementary Figures

## Abbreviations:

AR: after-ripening
CS: cold stratification
Cvi: Cape Verde Island accession
DC_50_: days of cold stratification required to reach 50% of germination
DOG: delay of germination
DOxy_50_: days of EPPO storage required to reach 50% of germination
DSDS_50_: days of seed dry storage required to reach 50% of germination
EPPO: elevated partial pressure of oxygen
G_max_: maximum germination after 100 h of seed imbibition
L*er*: Landsberg *erecta* accession
NIL: near-Isogenic Line
pO_2_: partial pressure of oxygen
QTL: quantitative trait locus
RH: relative humidity
RIL: recombinant inbred line
ROS: reactive oxygen species.
