# Supplementary material for: Seed dormancy release accelerated by elevated partial pressure of oxygen is associated with DOG loci
Source: J Exp Bot. 2018 Apr 26;69(15):3601–8. doi: 10.1093/jxb/ery156 (PMC6022590; doi:10.1093/jxb/ery156)
Supplement: Supplementary Figures [file ery156_suppl_supplementary_figures.pdf]

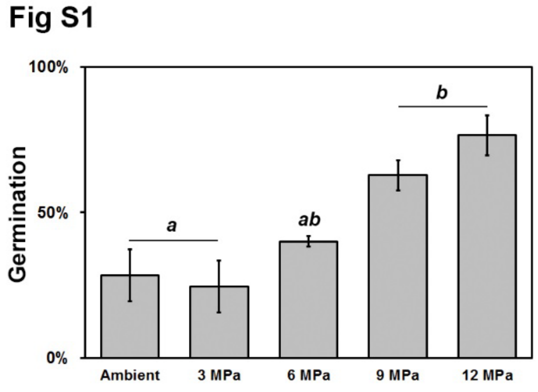

Fig. S1. Germination percentage after two weeks of storage under different EPPO treatments. Germination percentages of NIL*DOG1* after storage for two weeks at ambient pressure and 3, 6, 9 and 12 MPa air. Significant levels p=0.07, n=3.

Fig S2

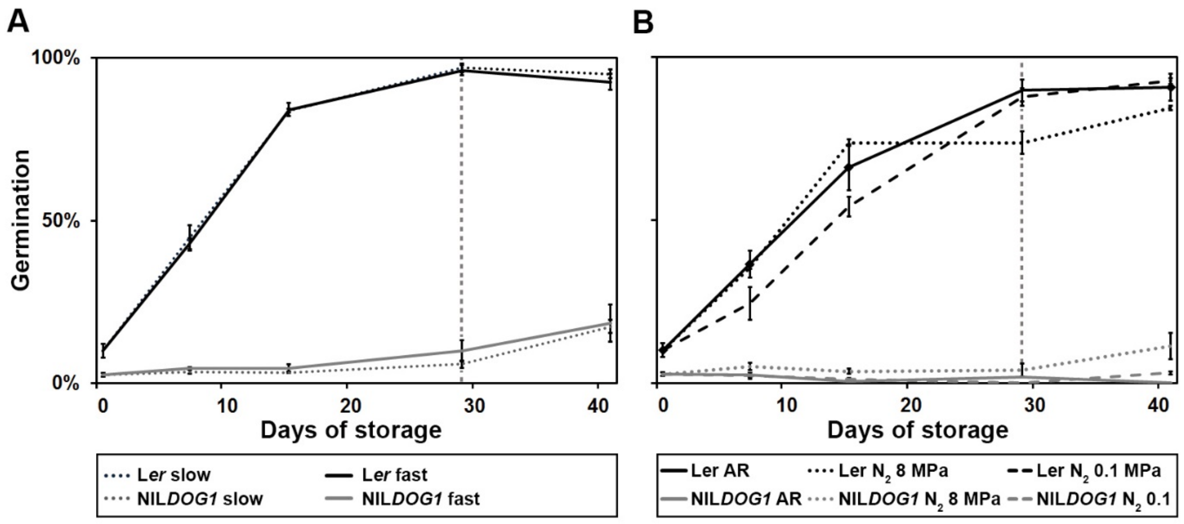

Fig. S2. Effect of the rate of pressure build up. A) Germination percentages of L*er* (black) and NIL*DOG1* (grey) after storage under 80 MPa N_2. Solid line: fast build-up of pressure (0.1 to 8 MPa in 2.5 minutes). Dotted line: slow pressure build-up (0.1 to 8 MPa in 25 minutes). B) Germination percentages of L*er* (black) and NIL*DOG1* (grey) after storage under AR, 0% oxygen at ambient (0.1 Mpa) and increased (8 MPa) pressure. Vertical dotted grey line indicates moment of pressure increase from 8 MPa to 20 Mpa (ambient pressure remains the same throughout the experiment).

Fig. S3. Frequency distributions and correlation plots for the QTL mappings of AR, EPPO and CS. A-C) Frequency distributions of the maximum germination percentages after the different storage periods of AR (A), EPPO (B) and CS (C) storage. D) Frequency distributions of the calculated DSDS50 (left) and DOxy50 (right). E) Correlation plots of the DSDS50 and DOxy50 per line (left) and per group of 7 genotypes (right). Based on the DSDS50, the genotypes were grouped in 23 groups of the same size, resulting in groups of seven genotypes. For each group the average was calculated and used to create the scatter plot. Table F indicates which germination time points correlate with each other. Table G summarizes the spatial pattern of the *DOG* loci.

Fig S3

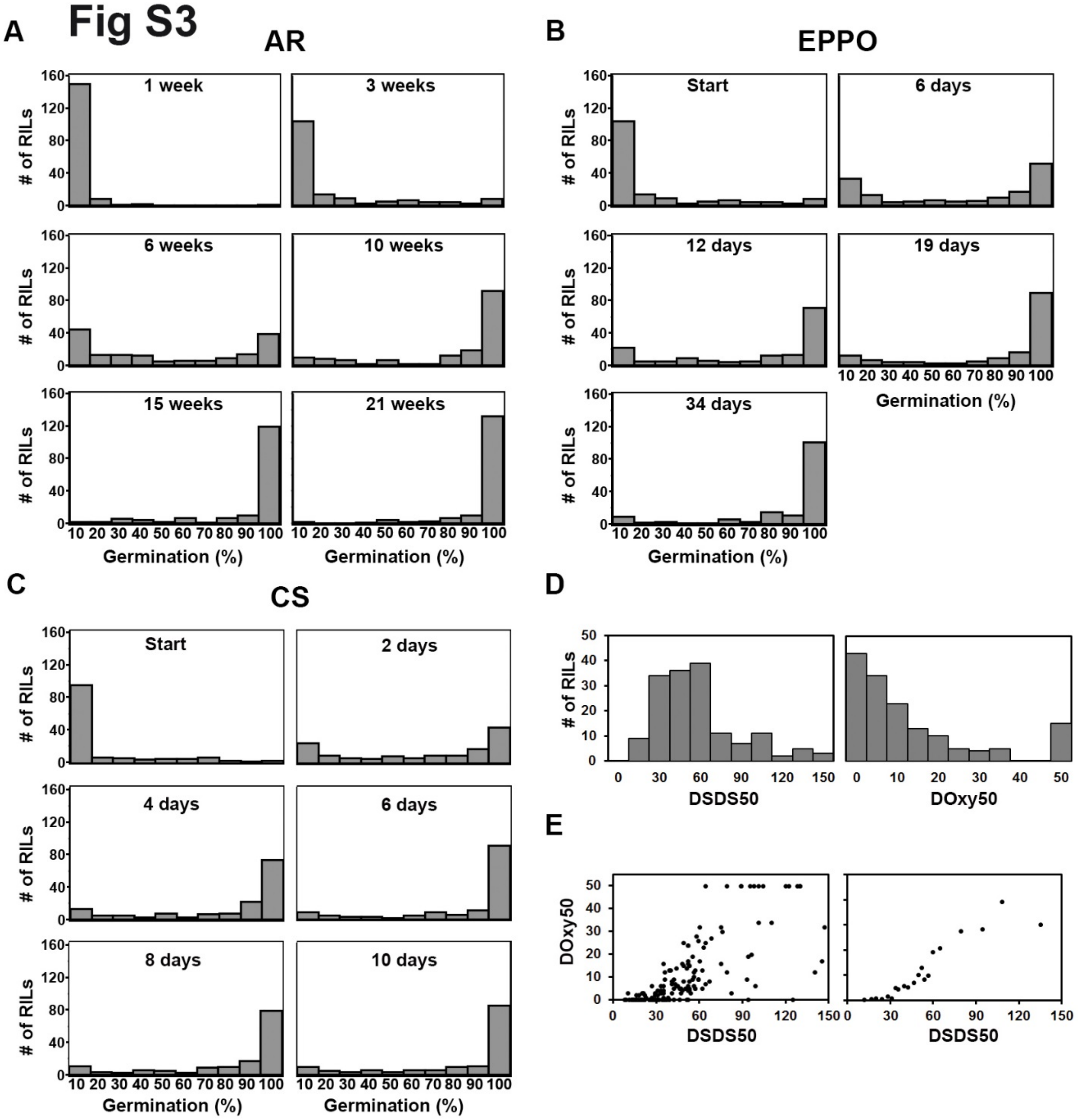

F

Pearson correlation coefficient between AR and EPPO at the different germination time points. Grey highlights indicate the highest correlation per time point.

	Start	6 days EPPO	12 days EPPO	19 days EPPO	34 days EPPO
1 week AR	0.309	0.264	0.218	0.174	0.139
3 weeks AR	0.633	0.529	0.460	0.387	0.327
6 weeks AR	0.588	0.716	0.700	0.651	0.575
10 weeks AR	0.362	0.581	0.667	0.755	0.784
15 weeks AR	0.231	0.386	0.469	0.582	0.639
21 weeks AR	0.138	0.238	0.302	0.407	0.450

G

Overview of the DOG QTL during EPPO and AR treatment.
"Sign" indicates significant detection of QTL. "---" QTL is not significant.

QTL	Treatment	3 weeks AR Start EPPO	6 weeks AR 6 Days EPPO	6 weeks AR 12 days EPPO	10 weeks AR 19 days EPPO	10 weeks AR 34 days EPPO
DOG2	AR	---	Sign	Sign	Sign	Sign
	EPPO	---	Sign	Sign	Sign	Sign
DOG3	AR	Sign	---	---	---	---
	EPPO	Sign	---	---	---	---
DOG6	AR	Sign	Sign	Sign	---	---
	EPPO	---	Sign	Sign	Sign	Sign
DOG5	AR	---	---	---	---	---
	EPPO	---	---	---	---	---
DOG4	AR	---	---	---	---	---
	EPPO	---	---	---	---	---
DOG1	AR	Sign	Sign	Sign	Sign	Sign
	EPPO	Sign	Sign	Sign	Sign	Sign
